# New approach to desensitization in solid organ transplantation-imlifidase

**DOI:** 10.3389/frtra.2022.951360

**Published:** 2022-11-10

**Authors:** Magdalena Durlik

**Affiliations:** Department of Transplantation Medicine, Nephrology and Internal Diseases, Medical University of Warsaw, Warsaw, Poland

**Keywords:** alloantibodies, sensitization, desensitization, imlifidase, kidney transplantation

## Abstract

The IgG-degrading enzyme derived from *Streptococcus pyogenes* is a recombinant cysteine protease of *S. pyogenes* produced in *Escherichia coli* that cleaves all four human subclasses of IgG with strict specificity. The proteolytic activity on IgG molecules prevents the occurrence of IgG-mediated antibody-dependent, cellular cytotoxicity and complement-mediated cytotoxicity, two processes that are critical for antibody rejection. The results from phase II studies demonstrated that desensitization with imlifidase represents a therapeutic strategy that can operationalize desensitization, allowing life-saving transplants from deceased donors (DD) and living donors (LD) to proceed in highly sensitized kidney transplant candidates with low risk of hyperacute rejection. Its action onset is rapid, allowing kidney transplantation from a deceased donor. Disadvantages of imlifidase include a quick reappearance of DSAs, which poses a risk of antibody-mediated rejection, the quick development of anti-Ides antibodies, which rules out repeated use of imlifidase and its IgG-degrading potential, limiting the use of therapeutic antibodies. Imlifdase received conditional approval on 26 August 2020 in the EU for desensitization treatment of highly sensitized adult kidney transplant patients with positive crossmatch against an available deceased donor.

Patients awaiting a kidney transplant may have anti-human leukocyte antigen (anti-HLA) antibodies in the blood, which reduces their chances of finding a suitable donor. The most common causes of sensitization are previous kidney transplantation, blood transfusions, pregnancy and sometimes, transiently, viral infections. It is estimated that about 30% of those on the waiting lists are sensitized patients. Sensitized patients with cPRAs (calculated Panel Reactive Antibodies) >20% account for about 15–30% of those on the waiting lists while highly sensitized patients with cPRAs > 80% account for 5–15%, of which 2–35% are patients with cPRA > 98%, i.e., are highly unlikely to be transplanted. Solid-phase tests allow identification of the specificity of antibodies produced by the recipient as well as their intensity (MFI). Depending on the transplant centre policy, patients with high DSA MFI (>3,000 or >5,000) are not eligible for final crossmatch with the donor (1).

Since highly sensitized patients wait for a kidney transplant for a very long time, transplant organizations have introduced programs promoting access to transplantation for this group of patients. In Eurotransplant, it is called Acceptable Mismatch. In the US, a new KAS (Kidney Allocation System) was introduced in 2014. The number of transplantations in highly sensitized patients has increased but access to this procedure is still limited.

According to USRDS Annual Report 2021 median waiting time for deceased donor id 51 months (2). Using the UNOS STAR file Schinstock et al. (3) analyzed prevalent active waiting-list cohort as of June 1, 2016, followed for 1 year. The overall transplantation rate was 18.9% (11 129/58769). Only 9.7% of candidates with a calculated panel reactive antibody ≥99.9% received a transplant. Nearly 4 years after KAS (6/30/2018), 1791 actively wait-listed candidates had a cPRA of ≥99.9% and 34.6% (620/1791) of these had ≥5 years of waiting time.The proportion and number of candidates who were on the waiting list for at least 5 years was 8.8% in the <80% CPRA group, 10.7% in the 80–89% CPRA group, 17% in the 90–98% cPRA group, and 20.9% in the 99% cPRA group (3).

In the EU, 40% of highly sensitized individuals fail to find a compatible recipient within 2 years. For the most sensitized patients, desensitization programs have been launched, aimed at eliminating or reducing anti-HLA levels. Typically, they involve using combined therapies: plasmapheresis, intravenous immunoglobulins (IVIGs) and rituximab, as well as bortezomib and eculizumab. Desensitization effectiveness is up to 80%, but a subset of recipients still fails to respond to this form of antibody-lowering therapy. Desensitization is even more effective when a potential recipient has a living donor, in which case, desensitization can be carried out immediately before transplantation. For a living donor, there is also the possibility to enroll on a kidney paired donation (KPD) program. In cases of waiting for a deceased donor, the desensitization program takes a number of weeks and its effect may be nullified by its prolonged waiting time (4). Marfo et al. (5) summarized 21 studies published between 2000 and 2010, involving 725 patients with donor-specific anti- HLA antibodies (DSAs) who underwent kidney transplantation with different desensitization protocols. All studies were single center and retrospective. The patient and graft survival were 95 and 86%, respectively, at a 2 year median follow-up. Despite acceptable short-term patient and graft survivals, acute rejection rate was 36% and acute antibody-mediated rejection rate was 28%, which is significantly higher than in non-sensitized patients (5).

In 2002, cysteine protease isolated from *Streptococcus pyogenes* (IdeS, imlifidase) was identified, which eliminates IgGs from the blood (6). In the *in vitro* settings, IdeS also removes circulating B cell receptors by inhibiting antigen-specific B cell response (7).

IdeS has been used for desensitization of highly sensitized patients awaiting kidney transplantation. The enzyme can cleave heavy chains of all human IgG subclasses but has no effect on other immunoglobulins. The IgG cleaving results in the elimination of Fc-dependent effector functions, including complement-dependent cytotoxicity (CDC) and antibody-dependent cellular cytotoxicity (ADCC) (8). By cleaving all IgGs, imlifidase reduces DSA concentration and enables organ transplantation. Imlifidase cleaves not only plasma IgGs, but also the entire IgG pool, including extravascular IgGs. An immediate effect, i.e., absence of circulating IgGs within 4–6 h of administration, is one advantage of imlifidase. The recovery of endogenous IgG production takes place 2–3 days after administration, and after 2–3 weeks, IgGs are again the main Ig fraction, although the total IgG level is below the normal level for at least two more months (9).

The randomized, double-blind phase 1 PK/PD study involved 20 healthy volunteers. The study drug was administered intravenously at increasing doses. IdeS eliminated IgGs from plasma within minutes of administration, and its peak effect persisted for 6–24 h. Subsequently, after 2–3 days, IgG gradually reappeared in the bloodstream. IdeS half-life was 4.9 (±2.8) h for the 0.24 mg/kg dose. The medicine proved safe and caused no serious adverse reactions or toxicities with increasing doses. The complete, rapid but temporary, IgG elimination has a therapeutic potential in diseases caused by IgG antibodies (10).

Lorant et al. (11) published the results of a phase 2 study assessing the safety, immunogenicity, pharmacokinetics and efficacy of imlifidase in highly sensitized patients with stage five chronic kidney disease. Eight patients with a mean cPRA of 64% received two increasing doses for two consecutive days [0.12 mg/kg b.w. x 2 (*n* = 3); 0.25 mg/kg x 1 (*n* = 3); 0.25 mg/kg x 2 (*n* = 2)]. IgG degradation was seen in all patients; serum IgG levels were <1% within 48 h and remained low to Day 7. MFI values for C1q-binding class I and II anti-HLA were significantly reduced. Anti-IdeS antibodies developed 1 week after treatment and reached a peak after 2 weeks. During the study, one of the patients was offered a deceased donor kidney transplantation, but crossmatch was positive. Following imlifidase administration, anti-HLA elimination was observed and a successful kidney transplantation was performed. The authors emphasize that in sensitized patients, imlifidase is effective, safe and well-tolerated. Furthermore, it provides a 7-day time window in which anti-HLAs are not detectable and the patient has a chance of successful HLA-incompatible donor kidney transplantation (11).

A similar single-center study was conducted by Lonze et al. (12) IdeS was administered to seven potentially highly sensitized (cPRA 98–100%) kidney recipients (five from a deceased donor and two from a living donor), with a positive crossmatch 24 h before transplantation. All crossmatches following imlifidase administration were negative and all patients underwent kidney transplantation. In three patients, DSA levels increased rapidly, resulting in antibody-mediated rejection (ABMR) 8, 10, and 27 days after transplantation, which was managed by standard therapy. In three recipients, the graft function was delayed. DGF did not occur in living donor recipients, only in 3 kidney recipients from deceased donors.

No serious adverse reactions were observed, and all recipients had a functioning graft after a mean of 235 days of follow-up. In the opinion of the authors, imlifidase is a new, effective desensitization option that provides patients with a chance of receiving life-saving kidney transplant (12).

The results of the first two-center phase 1/2 study were published by Jordan et al. (13, 14). The studies were conducted independently in Sweden and the USA (NCT02224820, NCT02426684, and NCT02475551). They assessed imlifidase efficacy in the desensitization of highly sensitized patients and the feasibility of HLA-incompatible donor kidney transplantation. The medicine was administered to 25 highly sensitized recipients (11 in Sweden and 14 in Los Angeles) prior to transplantation. Maintenance immunosuppression included tacrolimus, mycophenolate mofetil, and glucocorticosteroids. As induction, Swedish recipients received horse ATG while the American received alemtuzumab. In the US, the patients also received intravenous immunoglobulins (IVIGs) on post-transplantation days 7–14 and rituximab on post KTx days 14–21 as ABMR prophylaxis. All the recipients received antibiotic prophylaxis in the period of IgG absence. At the time of transplantation, no recipients had IgG anti-HLA antibodies. ABMR was seen in 10 recipients: seven (50%) in the US and three (27%) in Sweden from 2 weeks to 5 months after transplantation. All responded to treatment. Graft loss was observed in one patient in US cohort 1/14 (hyperacute rejection, non HLA IgA and IgM antibodies), no graft loss was observed in Swedish cohort 0/11. DSA levels rose at 1–2 week in both US and Swedish group. U.S. cohort had fewer patients with rebound and lower levels of HLA antibodies after treatment with IdeS but precise numerical data are not presented. DSA remained absent up to 12 months after transplant in most patients which was attributed to the use of IVIG and rituximab to prevent antibody rebound in the United States study.

Thirty-eight serious adverse reactions were reported in 15 patients (five of which were considered likely related to IdeS). The study demonstrated that imlifidase was effective in desensitization; 24/25 patients underwent successful kidney transplantation; after 6 months of follow-up, the mean eGFR was 58 ± 30 mL/min/1.73 m^2^ (13).

Another single-arm, open-label phase 2 study was aimed at assessing the efficacy and safety of imlifidase in converting a positive crossmatch test to negative, enabling sensitized patients to receive a living or deceased donor transplant. Study 15-HMedIdeS-06, Highdes, was conducted at five transplant centers (Cedars-Sinai Medical Center, Los Angeles; The Johns Hopkins Hospital, Baltimore; New York University Langone Health, New York; Uppsala University Hospital, Uppsala; Hôpital Necker, Paris) between September 2016 and July 2018 (EudraCT Number: 2016-002064-13); it involved 19 recipients (13 from deceased donor transplants), with a mean cPRA value of 99.83% (range: 77.31–100.0%) and the cut-off MFI value of 3,000. The primary endpoint was negative crossmatch within 24 h. The follow-up period was 6 months. Secondary endpoints included DSA levels, graft kidney function and drug pharmacokinetics and pharmacodynamics. Among transplant recipients, in 89.5% (17/19) crossmatch conversion was seen within 24 h; 18 patients received a kidney transplant. All recipients received induction with glucocorticoid pulse (3x, first dose intraoperatively), 4 patients received equine antithymocyte serum 15 mg/kg IV for 4 days and 14 patients received one dose of alemtuzumab 30 mg IV (Campath) on Day 4. In addition, IVIGs at 2 g/kg b.w. (max. 14,0 g) on Day 7 and rituximab (anti-CD20) 1 g IV on Day 9 were administered.

Maintenance immunosuppression included tacrolimus, MMF and glucocorticosteroids. To reduce the risk of infections, all the recipients received prophylactic antibiotic therapy until IgG levels increased. DSA reappeared on days 3–14 after imlifidase administration in all but two recipients. Patient survival was 100% at 6 months and graft survival was 88.9%; two recipients lost the graft (deceased donor) due to primary non-function (not ABMR). In 7 (38.9%) of patients, early ABMR was diagnosed within 2–19 days of KTx. ABMR was treated with the standard of care including PE, IVIgs, glucocorticoids and optimization of maintenance immunosuppression. In addition, some patients received rituximab (*n* = 2), eculizumab (*n* = 3) or bortezomib (*n* = 2), and some underwent spleen embolization (*n* = 1) or splenectomy (*n* = 1). Ten recipients underwent a follow-up kidney biopsy after 6 months of follow-up. Among these, seven had DSAs at the end of the study (MFI = 8,000–18,000). Two cases of active ABMR and one case of chronic ABMR were reported. Other biopsies showed no evidence of ABMR. Mean eGFR in recipients with active graft at 6 months was 47 mL/min/1.73 m2 (21–92). The authors emphasize the feasibility of effective sensitization of the most sensitized recipients *(calculated panel-reactive antibodies–*99.83%) and successful transplantation thanks to the use of imlifidase (14).

Kjellman et al. (15) summarized 3 year results of four phase 2 studies (13-HMedIdeS-02, 13-HMedIdeS-03, 14-HMedIdeS-04 and 15-HMedIdeS-06) conducted at six transplant centers in the US and Europe. They analyzed data from 39 recipients with a mean cPRA value of 99.62% and a positive crossmatch, who received imlifidase before transplantation. The incidence of antibody-mediated rejection was 38% (15 pts), and in the majority of cases, it occurred in the first post-KTx month. ABMR was found to be correlated with higher DSA levels before and after imlifidase administration. Patient survival at 3 years was 90%. Three recipients died during follow-up: all between post-KTx Month 6 and 12. Survival was 85% in the ABMR group and 94% in the non-ABMR group. The 3 year graft survival rate was 84%. Three grafts were lost within 6 months–one due to hyperacute non-IgG-mediated rejection and two due to primary non-function. Two other grafts were lost 2 and 3 years after KTx–one due to reduced immunosuppression associated with infection and the other due to non-adherence to the immunosuppression regimen. The 3 year graft survival rate in recipients with ABMR vs. without ABMR was 93 vs. 77%; the mean eGFR was 55 mL/min/1.73 m^2^, including 49 mL/min/1.73 m^2^ vs. 61 mL/min/1.73 m^2^ in the ABMR and non-ABMR group, respectively. Three year follow-up data show that patients desensitized with imlifidase achieve similar results as other highly sensitized patients undergoing desensitization. Imlifidase appears to be a promising therapeutic option allowing for transplantation in patients with a significant immune barrier, even those untransplantable, with a cPRA value of 99.9% (15).

Summary of published clinical trials of imlifidase use in kidney transplant recipients is presented in Table 1.

**Table 1:** Key clinical studies of imlifidase in kidney transplant recipients.

**Study**	**Number of patients**	**Primary endpoint**	**Secondary endpoint**	**Induction immunosuppression**	**Results**
Phase 1 Winstedt et al. (10)	20 healthy subjects	Dosing	PK, immunogenicity PD, efficacy	NA	Complete, rapid, but temporary removal of IgG. Safe with no serious adverse events
Phase 2 Lorant et al. (11)	8 highly sensitized	safety, immunogenicity, pharmacokinetics and efficacy	HLA reduction (MFI <1,100 in SAB assay)	NA	Circulating IgG reduced to <1% within 48 h
Phase 2 Lonze et al. (12)	7 highly sensitized	Crossmatch conversion	NA	Methylprednisolone iv for 5 days, Alemtuzumab on postoperative day 4. Intravenous immune globulin was given either as a single dose on POD7, or in 2 divided doses on POD7+/-1, and Rituximab 1,000 mg, IV was given on POD9	100% crossmatches converted from positive to negative 100% PS and GS survival at 6 months 43% rate of ABMR
Phase 1/2 Jordan et al. (13)	25 highly sensitized recipients (11 in Sweden and 14 in US	Safety and efficacy	Renal function DSA day 0–180	Swedish recipients received horse ATG while the American received alemtuzumab. In the US, the patients also received intravenous immunoglobulins (IVIGs) on post-KTx days 7–14 and rituximab on post KTx days 14–21	24/25 patients transplanted 10 ABMR 1 graft loss (attributed to IgM/IgA)
Phase 2 HIGHDES Jordan et al. (14)	18 highly sensitized	Negative crossmatch within 24 h	DSA levels, graft kidney function and drug pharmacokinetics and pharmacodynamics	glucocorticoid pulse (3x, first dose intraoperatively), 4 patients received equine antithymocyte serum 15 mg/kg IV for 4 days and 14 patients received one dose of alemtuzumab 30 mg IV on Day 4. In addition, IVIGs at 2 g/kg b.w. (max 140,0 g) on Day 7 and rituximab 1 g IV on Day 9	89.5% of XM converted from positive to negative within 24 h 100% patient survival and 89% graft survival at 6 months. DSA rebound seen between 3 and 14 days post-imlifidase. 39% rate of ABMR.
Three years results of four phase two studies (13-HMedIdeS-02, 13-HMedIdeS-03, 14-HMedIdeS-04 and 15-HMedIdeS-06) Kjellman et al. (15)	39 highly sensitized	Long-term follow up	NA	NA	ABMR-38% PS-90% GS-84%

*DSA, donor specific antibodies; ABMR, antibody mediated rejection; GS, graft survival; PS, patient survival; PK, drug pharmacokinetics; PD, drug pharmacodynamics*.

On the basis of phase 2 studies, in August 2020, imlifidase was conditionally approved in the European Union (16). Imlifidase (Idefirix^®^, Hansa Biopharma AB) is indicated for desensitization treatment of highly sensitized adult kidney transplant recipients with positive crossmatch against the available deceased donor. The use of Idefirix should be reserved for patients unlikely to be transplanted under the existing kidney allocation system, including prioritization programs for highly sensitized patients.

There are several adverse phenomena associated with the use of imlifidase, which must be taken into account in the management of patients and which require further solutions. One is a quick reappearance of DSAs, which poses a risk of antibody-mediated rejection and, consequently, intensification of immunosuppression, which in turn may entail the risk of infections and other complications. Therefore, the selection of patients for treatment with imlifidase should be extremely careful and not include elderly patients and those with multiple comorbidities (diabetes, atherosclerosis, advanced cardiovascular diseases). The second problem is the quick development of anti-Ides antibodies, which rules out repeated use of imlifidase. Lorant et al. (11) in his study reported an increase in anti-IdeS IgG concentrations at day 7 after treatment in all patients. The peak serum concentration occurred on day 14 Substantial individual variation was observed for the magnitude of anti-IdeS response, with a median peak concentration of 875 mg/L, ranging between 190 and 1,000 mg/L. On day 64, the median concentration in serum had decreased to 120 mg/L (range 87–280 mg/L). Imlifidase, with its IgG-degrading potential, limits the use of therapeutic antibodies. Only equine ATG and eculizumab can be used immediately after imlifidase administration. Infusions of IVIGs after 12 h at the earliest, while alemtuzumab, basiliximab, rituximab and rabbit ATG after 4 days at the earliest. Belatacept can be administered safely after seven days (8, 17). Recently Bockermann et al. (18) showed that imlifidase generated single-cleaved IgG may persist in circulation and can cause positive assay results equivalent to intact IgG in clinical assays. Therefore, complete IgG cleavage after imlifidase treatment is essential to allow correct decision-making in relation to transplant eligibility (18).

The initial clinical experience with imlifidase to date includes 39 highly sensitized renal transplant recipients. We need larger patients series and long-term results. It seems that imlifidase is not for use in other solid organ transplant recipients. Liver is not immunogenic organ and HLA matching and crossmatch are not necessary to perform before transplantation. Regarding heart and lung transplantation due to short ischemia time (4–6 h without machine perfusion) there is no time for waiting for the result of crossmatch. Some centers perform retrospective crossmatch or only virtual crossmatch. Management of imlifidase needs first positive crossmatch and second crossmatch within 24 h after drug administration (6 h or longer). Imlifidase seems to be effective in the treatment of antibody mediated rejection. Randomized study enrolling patients with ABMR is ongoing (NCT03897205). Advantages and disadvantages of imlifidase are summarized in Table 2.

**Table 2:** Advantages and disadvantages of imlifidase.

**Advantages**	**Disadvantages**
Safety and efficiently cleaves IgG	Transient effect, recurrence of DSA at 7–14 days post-dosing
Effective in the most highly sensitized patients with cPRA > 98%	Risk of ABMR Intensification of immunosuppression-risk of infections
Effect in 4–6 h, capability of_transplantation from deceased donor	Quick development of anti-Ides antibodies
	IgG-degrading potential, limits the use of therapeutic antibodies

*DSA, donor specific antibodies; ABMR, antibody mediated rejection; cPRA, calculated panel reactive antibodies*.

In conclusion, imlifidase is a new therapeutic option for desensitizing highly sensitized patients awaiting kidney transplantation. It is effective and safe and its action onset is rapid, allowing kidney transplantation from a deceased donor. However, imlifidase does not cause long-term inhibition of antibody production. Anti-HLA antibodies recur quickly, which is why patients receive induction therapy, IVIGs and adequate maintenance immunosuppression to prevent ABMR. Nevertheless, ABMR is observed in 40% of recipients, mostly responders. Imlifidase is used only once because circulating anti-imlifidase antibodies are formed as soon as 1 week after administration.

## Author contributions

The author confirms being the sole contributor of this work and has approved it for publication.

## Conflict of interest

The author declares that the research was conducted in the absence of any commercial or financial relationships that could be construed as a potential conflict of interest.

## Publisher's note

All claims expressed in this article are solely those of the authors and do not necessarily represent those of their affiliated organizations, or those of the publisher, the editors and the reviewers. Any product that may be evaluated in this article, or claim that may be made by its manufacturer, is not guaranteed or endorsed by the publisher.
